# Dissociation between Cerebellar and Cerebral Neural Activities in Humans with Long-Term Bilateral Sensorineural Hearing Loss

**DOI:** 10.1155/2019/8354849

**Published:** 2019-03-27

**Authors:** Xiao-Min Xu, Yun Jiao, Tian-Yu Tang, Jian Zhang, Chun-Qiang Lu, Ying Luan, Richard Salvi, Gao-Jun Teng

**Affiliations:** ^1^Jiangsu Key Laboratory of Molecular Imaging and Functional Imaging, Department of Radiology, Zhongda Hospital, Medical School, Southeast University, Nanjing, China; ^2^Center for Hearing and Deafness, University at Buffalo, Buffalo, NY 14214, USA

## Abstract

Abnormal neural activity in the cerebellum has been implicated in hearing impairments, but the effects of long-term hearing loss on cerebellar function are poorly understood. To further explore the role of long-term bilateral sensorineural hearing loss on cerebellar function, we investigated hearing loss-induced changes among neural networks within cerebellar subregions and the changes in cerebellar-cerebral connectivity patterns using resting-state functional MRI. Twenty-one subjects with long-term bilateral moderate-to-severe sensorineural hearing loss and 21 matched controls with clinically normal hearing underwent MRI scanning and a series of neuropsychological tests targeting cognition and emotion. Voxel-wise functional connectivity (FC) analysis demonstrated decreased couplings between the cerebellum and other cerebral areas, including the temporal pole (TP), insula, supramarginal gyrus, inferior frontal gyrus (IFG), medial frontal gyrus, and thalamus, in long-term bilateral sensorineural hearing loss patients. An ROI-wise FC analysis found weakened interregional connections within cerebellar subdivisions. Moreover, there was a negative correlation between anxiety and FC between the left cerebellar lobe VI and left insula. Hearing ability and anxiety scores were also correlated with FC between the left cerebellar lobe VI and left TP, as well as the right cerebellar lobule VI and left IFG. Our results suggest that sensorineural hearing loss disrupts cerebellar-cerebral circuits, some potentially linked to anxiety, and interregional cerebellar connectivity. The findings contribute to a growing body showing that auditory deprivation caused by cochlear hearing loss disrupts not only activity with the classical auditory pathway but also portions of the cerebellum that communicates with other cortical networks.

## 1. Introduction

According to the World Health Organization (WHO), hearing loss (HL) of sensorineural origin affects approximately 466 million people (5% of the world's population) and ranks as the fifth leading age-related disability [1]. It is estimated that by 2050, over 900 million people will have disabling HL. Sensorineural hearing loss (SNHL) typically results from damage to outer and inner hair cells, spiral ganglion neurons, and auditory nerve fibers which project to the central auditory system [2, 3]. Recent studies have shown that SNHL alters neural activity not only in the central auditory pathway but also in the nonauditory structures such as the hippocampus, supramarginal gyrus (SMG), occipital gyrus, calcarine cortex, and prefrontal cortex [4–7]. The loss of sensory information could contribute to distractibility [8], cognitive decline [9], loss of independence [10], and a range of emotional reactions [11].

The cerebellum has traditionally been thought of as being involved in motor control; however, emerging evidence indicates that the cerebellum also plays important roles in sensory, cognitive, and emotional processes [12–14]. All acoustic information transduced in the cochlea is transmitted through the auditory nerve to the cochlear nucleus in the brainstem; some of this information is relayed to the cerebellum [15–17] while some regions of the cerebellum project back to the cochlear nucleus [18]. The cerebellum also projects to other nuclei in the central auditory pathway [19] while parts of the descending auditory pathway project to the cerebellum [20]. Electrophysiological studies in animals and functional imaging studies in human have revealed auditory-evoked activity in portions of the cerebellum [21–28]. A meta-analysis of 15 human imaging studies indicated that portions of the cerebellum play a purely sensory role in auditory processing [29].

From a global perspective, the cerebellum lies within independent sets of closed cortico-cerebellar loops, including “the motor loop” and “prefrontal loop,” which provide the structural and functional foundations linking the cerebellum to higher-order behaviors [30]. Abnormal volume, perfusion, and neural activity in the cerebellum of individuals were indicated in various psychiatric disorders, including schizophrenia [31], depression [32], anxiety [33], and neurodegenerative dementia [34]. The cerebellum receives dopaminergic inputs from the ventral tegmentum, a region associated with cerebellar neuroplasticity. Thus, drug-induced dopaminergic changes in emotion have been linked to cerebellar abnormalities [35]. Taken together, these results suggest that SNHL and resulting auditory deprivation could disrupt cerebellar function and contribute to changes in global higher-order emotional function such as anxiety or depression [36]. Indeed, a number of recent surveys have reported higher levels of anxiety and depression among individuals with HL [37–39].

Functional imaging and electrophysiological studies using an animal model of drug-induced HL and tinnitus identified an auditory-cerebellar network, in which some neurons in the parafloccular lobe of the cerebellum (PFL) and vermis respond to sound through direct and indirect connections with neurons in the cochlear nucleus (CN), inferior colliculus (IC), and auditory cortex (ACx) [25, 28, 40–42]. Structural MRI studies revealed increased cerebellar grey matter volume in early deafness [43]. A connectome-level analysis which mapped selected components revealed decreased functional connections in subcortical and cerebellar networks in long-term unilateral HL [44]. While there is a growing awareness that the cerebellum may be directly or indirectly involved in SNHL and its associated comorbid symptoms such as tinnitus, hyperacusis, cognition, depression, and anxiety, there is a paucity of evidence from functional neuroimaging studies linking disturbances in functional connectivity (FC) within the cerebellum and neural networks linking the cerebellum with other regions of the brain. To address this issue, we carried out a resting-state FC study in which we hypothesized that [1] SNHL disrupted connectivity within the cerebellum and cerebellar-cerebralconnectivity and [2] some features of disrupted FC were linked to comorbid emotional characteristics in the SNHL group.

## 2. Materials and Methods

### 2.1. Subjects

**Figure 1:**
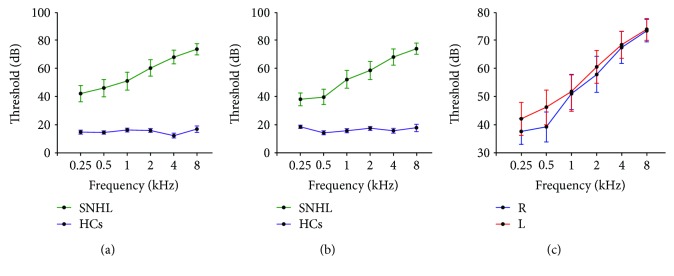
Pure tone audiometry (PTA) test of the long-term bilateral moderately severe sensorineural hearing loss (SNHL) patients and healthy controls (HCs). (a) Mean (±SEM) PTA of right ears of the SNHL group and HCs. Compared to HCs, thresholds are significantly higher in right ears of the SNHL group at 0.25, 0.5, 1, 2, 4, and 8 kHz, *p* < 0.001. (b) Mean (±SEM) PTA of left ears of the SNHL group and HCs. Compared to HCs, thresholds are significantly higher at every frequency, *p* < 0.001. (c) There was no significant difference between left and right ears in long-term bilateral SNHL subjects, *p* > 0.05.

SNHL has been ranked into five severity levels in the Global Burden of Disease (GBD) 2013 (i.e., moderate: 35-49 dB, moderately severe: 50-64 dB, severe: 65-79 dB, profound: 80-94 dB, and complete ≥ 95 dB). In the present study, twenty-one long-term bilateral, moderately severe SNHL subjects and 21 normal hearing subjects matched for age, sex, body mass index (BMI), and education level were recruited from the clinic of the E.N.T. Department of Zhongda Hospital and local community. All subjects in the two groups were right-handed and had completed at least 8 years of education. Diagnosis of long-term SNHL was based on pure tone audiometry (PTA) at frequencies of 0.25, 0.5, 1, 2, 4, and 8 kHz. Detailed information regarding the assessment of the subjects is shown in Table 1 and Figure 1.

None of the participants were excluded due to excessive head motion which needed to be less than 2.0 mm in translation and 2.0 degrees in rotation. Inclusion criteria were average PTA values of >25 dB [45], duration > 1 year, and no conductive and/or congenital HL. Participants were excluded from the present study if they [1] suffered from Meniere's disease, pulsatile tinnitus, vestibular diseases, vertigo, Parkinson's disease, Alzheimer's disease, brain injury, or MR image contraindications (e.g., cochlear implants, pacemakers, or prosthetic valves) or [2] had a history of sign language, hearing aids, alcohol or cigarette abuse, stroke, tumor in brain lesions, or neurologic disorder.

### 2.2. Ethical Considerations

Written informed consent was provided by all subjects before their participation in the study. All experimental procedures were approved by the ethics committee of Zhongda Hospital, Southeast University (2016ZDSYLL031.0), and in accordance with the declaration of Helsinki [46].

### 2.3. Neuropsychological Tests

All participants underwent a detailed battery of neuropsychological tests. Mini-Mental State Examination (MMSE), Auditory Verbal Learning Test (AVLT), and Symbol Digit Modalities Test (SDMT) targeting cognitive function. The Self-Rating Anxiety Scale (SAS) and Hamilton Depression Scale (HAMD) were used to evaluate mental status.

### 2.4. MR Imaging Data Acquisition

The functional and structural imaging data were acquired at the Radiology Department of Zhongda Hospital using 3.0 Tesla MRI (Siemens MAGENETOM Trio, Erlangen, Germany) with a standard head coil. Foam padding and earplugs (Hearos Ultimate Softness Series, USA) were used to attenuate scanner noise by approximately 32 dB. Subjects were instructed to stay still, keep their eyes closed, remain awake, and avoid thinking of anything in particular during the scan. The whole-brain fMRI dataset which took about 8 min and 6 sec to collect was based on BOLD signals. Images were obtained axially using a gradient echo-planar imaging (EPI) sequence. Parameters were as follows: 36 slices, volume = 240, repetition time (TR) = 2000 ms, echo time (TE) = 25 ms, section thickness = 4 mm, gap = 0 mm, field of view FOV = 240 mm × 240 mm, acquisition matrix = 64 × 64, and flip angle (FA) = 90°. We then used 3D MPRAGE sequence to acquire high-resolution (1 mm^3^) T1-weighted images (sections = 176, TR/TE = 1900/2.48 ms, inversion time = 900 ms, FA = 90, FOV = 256 mm × 256 mm, and acquisition matrix = 256 × 256).

### 2.5. Functional Data Processing

Data analysis was conducted using Data Processing Assistant for Resting-State fMRI programs (http://rfmri.org/DPARSF), which is based on statistical parametric mapping (SPM8; http://www.fil.ion.ucl.ac.uk/spm). The preprocessing steps were as follows: (a) removing the first 10 time points for signal equilibrium of initial magnetic resonance signals and adaption of the subjects to the scanner; (b) slice timing adjustment; (c) realignment for head motion correction; (d) segmentation and normalization to the Montreal Neurological Institute (MNI) template by linear transformation (resampling to 3 × 3 × 3 mm^3^ isotropic voxels); (e) regressing 6 motion parameters, 6 temporal derivatives, and 12 corresponding squared items using the Friston-24 parameter model to minimize the effect of head motion [47–49]; (f) smoothing using a 6 mm full-width half-maximum (FWHM) Gaussian kernel; and (g) detrending and band-pass filtering (0.01-0.08 Hz) to reduce the effect of low-frequency drift and high-frequency noise.

### 2.6. Statistical Analysis

#### 2.6.1. Clinical Data

Demographic and clinical variables were compared between groups using SPSS software (version 18.0; SPSS Inc., Chicago, IL). An independent two-sample *t*-test was used for continuous variables (including age and BMI), and a chi-square test was used for categorical variables such as gender. A *p* value < 0.05 was considered statistically significant.

#### 2.6.2. Structural Data Analysis

Voxel-based morphometry (VBM) was computed to identify differences in brain anatomy using DARTEL VBM methods [50]. Structural data (three-dimensional T1-weighted images) were segmented into grey matter (GM), white matter (WM), and cerebrospinal fluid. The total brain parenchyma volume was counted as the sum of GM and WM volumes. Between-group comparison was then conducted to confirm whether SNHL influenced the brain volume.

#### 2.6.3. Functional Data Analysis

**Figure 2:**
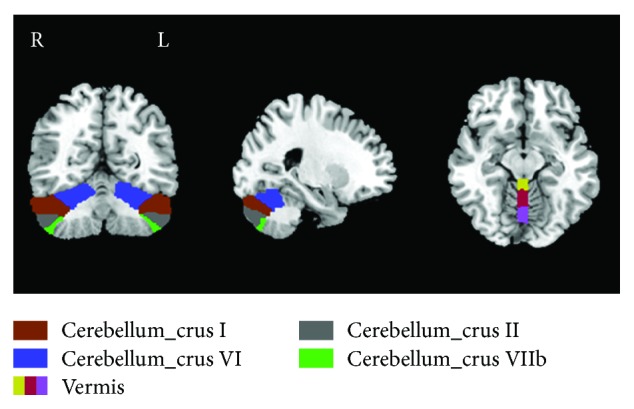
Nine subregions of the cerebellum including bilateral crura I (brown), II (grey), VI (indigo), and VIIb (green) and vermis (yellow/red/lavender). R: right hemisphere; L: left hemisphere. Slices shown for *X* = 27 mm, *Y* = -52 mm, and *Z* = -12 mm.

On the basis of previous findings [40], we separated the cerebellum into nine regions according to the Anatomical Automatic Labeling (AAL) atlas. The nine regions included the bilateral crus I, bilateral crus II, bilateral lobe VI, bilateral lobe VIIb, and vermis (Figure 2). Prior FC studies have provided evidence that these nine regions are involved with various aspects of sensorimotor function, the executive control network, the default mode network (DMN), the salience network, and the task-positive network [51–53]. The nine regions were resampled to a 3 × 3 × 3 mm^3^ standard space in order to enable extraction of time series from each subject's fMRI data. The resulting FC maps were transformed using Fisher's *z* to yield normally distributed data, and the average data for each subject was used for subsequent analyses. Each individual's *z* values were entered into random effect, one-sample *t*-test to identify the spatial distribution of each brain circuit region (*p* < 0.05 with family-wise error [FWE] correction). A two-sample *t*-test was performed to test for differences between SNHL patients and HCs. The mask was combined with the significant clusters of both groups. The significance threshold was set at *p* < 0.01 by FWE correction with age, sex, education level, and motion corrected. Furthermore, interregional seed-based matrices were also constructed among the nine chosen ROIs of the cerebellum, quantifying the unique functional relationships between each pair of ROIs. The uncorrected *p* value threshold was set at <0.01.

#### 2.6.4. Relationships between FC Data and Clinical Characteristics

To study the relationships between the FC results and the clinical variables, we extracted the signals from each significant cluster using REST software (http://www.restfmri.net). A Pearson correlation analysis was then conducted between mean *z* values of FC and each clinical feature (HL duration, neuropsychological tests, mean PTA of the left/right ear, and mean PTA of bilateral ears) using SPSS software. A *p* value < 0.05 was considered statistically significant.

## 3. Results

### 3.1. Demographic and Hearing Loss Characteristics

The two groups did not differ in age, gender, BMI, and education years (Table 1). SNHL patients had higher PTA thresholds in both the left and right ears compared to the normal hearing control group. In the SNHL group, the mean hearing threshold was >30 dB at 0.25, 0.5, 1, 2, 4, and 8 kHz. There was no significant difference in pure tone hearing thresholds between left and right ears in the SNHL group (Figure 1(c)). Consistent with GBD 2013, patients included in our study reached a moderately severe degree of HL because the mean PTA thresholds of both ears was 56.8 dB (±20.7 dB). No significant differences were observed between the two groups on the MMSE, SDMT, and AVLT tests. However, there was a significant difference in SAS scores between the SNHL and control groups; SAS scores in the SNHL group were higher than those in the normal hearing control group. These results suggested that long-term auditory deprivation resulting from SNHL may increase anxiety.

### 3.2. Cerebellar-Cerebral Connectivity and SNHL

**Figure 3:**
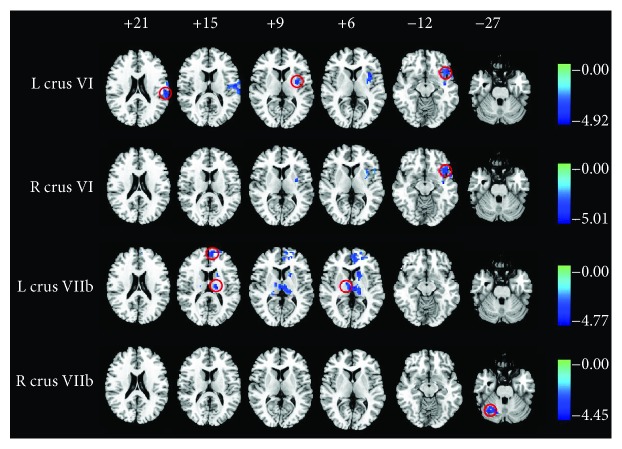
Whole-brain analysis of functional connectivity (FC) of nine cerebellum subregions. Decreased cerebellar-cerebral FC in SNHL patients compared to healthy controls indicated by colour (*p* < 0.01, family-wise error corrected). The colour heat map scale at the right shows *t* values. R: right; L: left.

No significant differences were found in GM and WM volume between the SNHL and normal hearing groups. However, there were significant differences between the SNHL group and HC group in terms of FC among the nine ROIs within the cerebellum (i.e., vermis and bilateral crura I, II, VI, and VIIb) and other brain regions as shown in Figure 3 and Table 2 (*p* < 0.01, FWE corrected). Compared to normal hearing controls, the SNHL group showed [1] reduced FC between left cerebellar crus VI and left temporal pole (TP), insula, and supramarginal gyrus (SMG), [2] reduced FC between the right cerebellar crus VI and left inferior frontal gyrus (IFG), [3] reduced FC between left cerebellar crus VIIb and left medial frontal gyrus (MFG)/bilateral thalamus, and [4] reduced FC between right cerebellar crus VIIb and right cerebellar crus I.

### 3.3. ROI-Wise Interregional Cerebellar Connectivity Analysis

**Figure 4:**
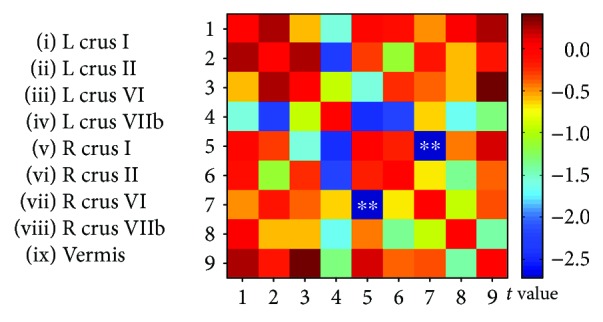
A matrix map of ROI-based interregional functional connectivity (FC) in the cerebellum of the SNHL group and HCs. Compared to HCs, the SNHL group showed a significant reduction of resting-state FC between right crus I and crus VI (*p* < 0.01, uncorrected). After FWE correction, no significant difference remained. The colour bar at the right shows *t* value. ^∗∗^*p* < 0.01.

A matrix map of rs-FC was constructed among the nine cerebellar subdivisions for the SNHL and HC groups, and a between-group comparison of the two matrices was performed to determine if there were significant differences between the two groups. As shown in Figure 4, rs-FC was significantly reduced in the SNHL group compared to the HC group between right crus I and right crus VI (*p* < 0.01, uncorrected); however, after FWE correction, these differences disappeared. No other significance between group differences in rs-FC was observed among the remaining cerebellar regions (Figure 4).

### 3.4. Correlation Analysis

**Figure 5:**
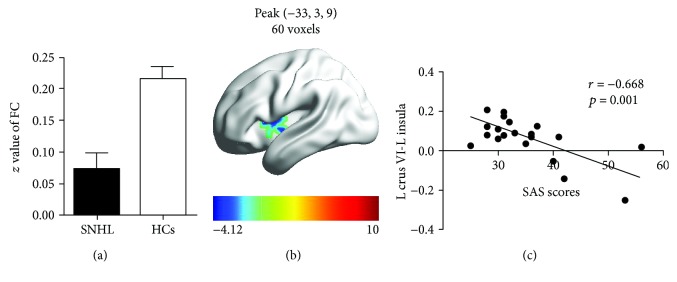
(a) Mean FC *z* values between left cerebellar crus VI and left insula. (b) Schematic of the left surface of the brain: coloured areas identify regions of reduced FC in the left insula from left crus VI. (c) FC *z*-scores between left crus VI and left insula versus the SAS anxiety score in the SNHL group. SAS scores were negatively correlated with *z*-score values of FC between left cerebellar crus VI and left insula (*r* = -0.668, *p* = 0.001). SNHL: sensorineural hearing loss; HCs: healthy controls; SAS: Self-Rating Anxiety Scale.

To assess the relationships between the fMRI results and the clinical data, Pearson correlation analyses were calculated. There were no significant correlations between cerebellar interregional FC measures and the clinical test scores. However, in the SNHL group, mean FC *z* values between left cerebellar crus VI and left insula were lower than in the control group (Figure 5(a)). Reduced FC *t*-scores in the left insula are shown in the heat map of the left hemisphere (Figure 5(b)). FC *z*-scores between left crus VI and left insula were negatively correlated with SAS anxiety scores (*r* = -0.668, *p* = 0.001) (Figure 5(c)).

**Figure 6:**
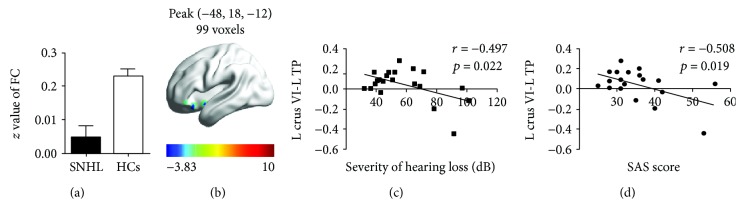
(a) Mean FC *z* values between left cerebellar crus VI and left TP. (b) Schematic of the left surface of the brain: coloured regions identify regions of reduced FC in left TP with left crus VI. (c) Voxel-wise correlations of FC *z*-score between left crus VI and left TP versus severity of hearing loss (dB). FC *z*-score between left cerebellar crus VI and left TP (*r* = -0.497, *p* = 0.022). (d) Voxel-wise correlations of FC *z*-score between left crus VI and left TP versus SAS anxiety score (dB) (*r* = -0.508, *p* = 0.019). SNHL: sensorineural hearing loss; HCs: healthy controls; TP: temporal pole; SAS: Self-Rating Anxiety Scale.

In the SNHL group, mean FC *z* values between left crus VI and left TP were lower than that in the control group (Figure 6(a)). The reduced FC *t*-score is illustrated with the heat map of the left hemisphere (Figure 6(b)). FC *z*-scores between the left crus VI and left TP were negatively correlated with severity of HL (*r* = -0.497, *p* = 0.022) (Figure 6(c)) and negatively correlated with SAS anxiety scores (*r* = -0.508, *p* = 0.019) (Figure 6(d)).

**Figure 7:**
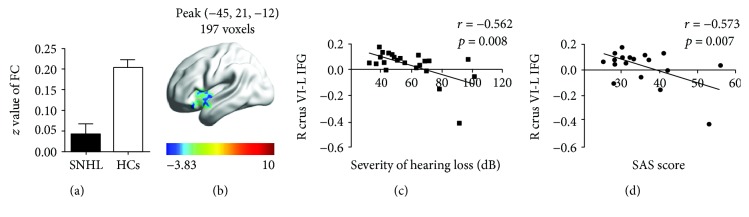
(a) Mean FC *z* values between right cerebellar crus VI and left IFG. (b) Schematic of the left surface of the brain: coloured regions identify regions of reduced FC in left IFG with right crus VI. (c) Voxel-wise correlations of FC *z*-score between right crus VI and left IFG versus severity of hearing loss (dB). FC *z*-score between right cerebellar crus VI and left IFG (*r* = -0.562, *p* = 0.008). (d) Voxel-wise correlations of FC *z*-score between right crus VI and left IFG versus SAS anxiety score (dB) (*r* = -0.573, *p* = 0.007). SNHL: sensorineural hearing loss; HCs: healthy controls; IFG: inferior frontal gyrus; SAS: Self-Rating Anxiety Scale.

Also, in the SNHL group, mean FC *z*-scores between left crus VI and left IFG were lower than that in the normal hearing control group (Figure 7). The reduced FC *t*-score in the IFG is indicated on the heat map of the left hemisphere (Figure 7(b)). FC *z*-scores between left crus VI and left IFG were negatively correlated with severity of HL (Figure 7(c)) (*r* = -0.562, *p* = 0.008) and also negatively correlated with SAS anxiety scores (Figure 7(d)) (*r* = -0.573, *p* = 0.007). In contrast, none of the correlations between FC values and the clinical scores were statistically significant in the normal hearing control group.

## 4. Discussion

FC MRI measures have been used to reveal the effects of hearing impairments on brain function [7, 25, 40]. To date, this is the first study identifying alterations of cerebellar-cerebral and interregional neural activities in long-term bilateral moderately severe SNHL patients. Disrupted cerebellum FC to auditory and nonauditory areas was observed in SNHL patients versus control subjects, as well as decreased connectivity within cerebellar subdivisions. It could be inferred from our research that SNHL disrupts the functional circuits leading to reorganization of higher-order control networks. Of note, these neural abnormalities in FC were correlated with the severity of HL and SAS scores, suggesting that cerebellum-related connectivity might be a candidate imaging marker for SNHL. Along with previous studies [15, 54], our findings provide support for the cerebellum as a crucial node in auditory and emotional processing.

The cerebellum has been found to play a role in tinnitus patients possibly through the gain-control mechanism [40]. Decreased levels of the benzodiazepine receptor (BDR) detected by ^123^I-iomazenil SPECT were shown in tinnitus, while BDR could enhance GABAergic neuronal transmission and inhibitory effect [55]. Nevertheless, the potential involvement of the cerebellum in SNHL remains to be further explored. Even though numerous somatosensory and sensory discrimination tasks have been applied to distinguish the function of the cerebellum in multimodal sensory information, few have concentrated on pure hearing tasks. Activation likelihood estimation meta-analysis [29] determined that the cerebellum was involved in fundamental calculations when acquiring sensory data in real-time, operating at the level of basic mechanisms of auditory sensory processing. Additionally, data from animals found shorter response latency in the cerebellum than the auditory cortex when responding to acoustic stimuli without emotion, cognition, or motor components [22, 42]. Our findings provided support for the cerebellum as a crucial node in auditory and emotional processing.

Both Voxel-wise and ROI-wise FC analyses found disassociations within the cerebellum: the right cerebellar crus I showed decreased connections with right cerebellar crura VI and VIIb (posterior lobes of the cerebellum). Clinical evidence suggests that the cerebellum functions in cognition and emotion patterns, lesions of the cerebellar posterior lobe could result in cognitive and affective impairments [56]. Independent component analysis (ICA) also confirmed the involvement of the cerebellum in the DMN and the frontoparietal network [57]. To our knowledge, this is the first demonstration of internal deficits of the cerebellum in SNHL, suggesting that SNHL may interrupt internal functioning of the cerebellum. Further study is needed to find the underlying pathology of cerebellar involvement in SNHL.

Weakened left cerebellar crus VI-SMG FC and left cerebellar crus VI-TP FC were observed in deafness. SMG (located inferior to the intraparietal sulcus and near the temporoparietal junction) can not only be activated by pure tones [58] but is also involved in the phonological processing and verbal working memory [59], as well as interfere with the function of the temporal lobe [60]. Interestingly, the left cerebellar crus VI-TP FC showed negative correlations with hearing ability and anxiety level. More than being an important node in traditional auditory cortex, the TP binds perceptual inputs to visceral emotional response [61] and strongly interconnected with amygdala [62], extending its role in emotion regulation [63]. The TP has been reported in anxiety disorders [64, 65], indicating its potential role as a biomarker for anxiety diagnosis and treatment. Moreover, the IFG and MFG showed a decline in FC to the cerebellum in our research. Parker et al. explored cerebellar-frontal interactions in schizophrenia animal models, and functional MRI revealed cerebellar activation in concurrence with frontal activation [66]. This suggests potential therapeutic targets in which cerebellar stimulation could promote recovery from dysfunctional frontal networks. Likewise, it was notable that IFG and MFG (parts of frontostriatal circuits, prominently projecting to the dorsomedial striatum [67]) are engaged in temporal action, particularly sound discrimination and language processing [68]. Our data are consistent with previous findings, showing that reduced connectivity in subjects with SNHL is negatively correlated with the severity of HL and SAS scores. We failed to find such a relationship between anxiety and HL (*r* = 0.240, *p* = 0.295), in contrast to other that found a positive correlation between severity of HL and anxiety [36]. Heterogeneity of subjects and accompanied symptoms (like tinnitus, depression, and stress) might contribute to this inconsistency.

In our study, the cerebellar subdivisions had weakened connectivity with the thalamus and insula, compared with the controls. The thalamus is considered as the main relay station of cerebral-cerebellar circuits, regulating information between deep cerebellar nuclei and cerebral cortex [69]. As a sensory hub, it relays inputs from sensory organs (like ears and eyes) to cerebral areas. An animal study demonstrated that auditory deprivation abolished acoustic activation in the thalamus and synaptic thalamocortical connections [70]. Lyness et al. observed increased microstructural measurements of mean diffusivity, radial diffusivity, and interrupted thalamocortical tracts in congenital deafness using diffusion MRI [71]. Based on anatomic connections between the thalamus and insula [72], the insula could also respond to internal/interoceptive and external (including acoustic) stimuli [73]. Sudden SNHL patients showed decreased FDG uptake in the insula, reflecting a “freezing” behavior during hearing traffic [74]. Considering its afferent and efferent connections with the anterior cingulate cortex and amygdala, the insula is thought to be involved in emotion processing [75], consistent with our data showing a correlation with SAS scores. The thalamus and insula are two key components of the reward neurocircuitry which links motivation with complex behaviors [76]. Auditory participats in the reward process with acoustic stimuli [77], and anxiety disorders often exert negative effect on the reward network [78]. More work is needed to determine how SNHL contributes to reward process and its correlation with SNHL-induced anxiety.

Several limitations of this study need to be acknowledged in addition to the relatively small population size of adult participants. First, the duration and medication of SNHL were quite variable in our study. In order to minimize the effects of confounding factors, further longitudinal studies with a larger sample size are needed to identify dynamic alternations in related brain areas and cognitive deficits in SNHL patients. Second, we cannot completely rule out the contribution of MR scanner noise which ranges from 103-113 dB SPL in 3 T MRI scanners [79], although we used earplugs and a sponge. Therefore, the confounds of MR imaging acoustic noise in nontask fMRI remain to be addressed since the FC changes may be due to internal and external stimulations. Third, the limitation is seed-based FC, which only investigates a limited number of interactions; hence, whole-brain connectivity analysis, such as graph theory-based network analysis, may better elucidate the key-region alterations in FC due to SNHL [80].

## 5. Conclusion

To conclude, this preliminary study is the first to discover widespread decreased cerebellar-cerebral connections and abnormal internal cerebellum function resulting from long-term bilateral moderately severe SNHL. Some of the weakened connectivity patterns were negatively correlated with severity of HL and neuropsychological scores. Our study provides novel insights into the role of the cerebellum and a better understanding of the neuropathology of cerebellar-cerebral interactions following sensory deprivation, proving a therapeutic target for SNHL-related emotional impairments.

## Figures and Tables

**Table 1:** Characteristics of long-term bilateral sensorineural hearing loss patients and healthy controls.

Characteristic	SNHL patients (*n* = 21)	Healthy controls (*n* = 21)	*p* value
Age (years)	54.2 ± 10.6	53.6 ± 8.8	0.826
Gender (male : female)	14 : 7	10 : 11	0.212
BMI	23.2 ± 3.1	23.3 ± 2.1	0.921
Education level (years)	11.2 ± 2.8	11.7 ± 3.4	0.590
Hearing loss duration (years)	8.2 ± 7.5	NA	NA
Mean PTA_left	58.2 ± 21.6	15.1 ± 5.0	<0.001^∗∗∗^
Mean PTA_right	55.5 ± 20.7	16.3 ± 4.5	<0.001^∗∗∗^
Mean PTA_bilateral	56.8 ± 20.7	15.7 ± 4.3	<0.001^∗∗∗^
MMSE	29.6 ± 0.8	29.7 ± 0.6	0.663
SAS	34.8 ± 8.0	29.1 ± 3.1	0.006^∗∗^
HAMD	6.4 ± 4.0	5.6 ± 8.9	0.706
SDMT	36.1 ± 12.0	38.2 ± 11.0	0.540
AVLT-5 min	6.0 ± 2.8	6.8 ± 1.5	0.273
AVLT-20 min	6.0 ± 2.8	6.3 ± 1.8	0.697

Data are represented as mean ± SD. ^∗∗^*p* < 0.01; ^∗∗∗^*p* < 0.001. SNHL: sensorineural hearing loss; BMI: body mass index; PTA: pure tone audiometry; MMSE: Mini-Mental State Examination; AVLT: Auditory Verbal Learning Test; SDMT: symbol digit modalities test; SAS: Self-Rating Anxiety Scale; HAMD: Hamilton Depression Scale.

**Table 2:** Decreased functional connectivity in SNHL subjects compared with healthy controls.

Seed region	Brain region	BA	MNI coordinate *x*, *y*, *z* (mm)	Peak *t*-score	Cluster size
L crus I	—	—	—	—	—
R crus I	—	—	—	—	—
L crus II	—	—	—	—	—
R crus II	—	—	—	—	—
L crus VI	L temporal pole	38	-48, 18, -12	-3.8264	99
	L insula	48	-33, 3, 9	-4.1166	60
	L supramarginal gyrus	42	-63, -27, 21	-4.3278	134
R crus VI	L inferior frontal gyrus	38	-45, 21, -12	-4.0291	197
L crus VIIb	L medial frontal gyrus	10	-9, 60, 15	-4.1873	173
	L thalamus	—	-18, -18, 15	-4.0176	260
	R thalamus	—	6, -15, 6	-4.109	194
R crus VIIb	R crus I	19	36, -66, -27	-3.9536	109
Vermis	—	—	—	—	—

BA: Brodmann area; MNI: Montreal Neurological Institute; L: left; R: right.

## Data Availability

The data used to support the findings of this study are available from the corresponding author upon request.

## References

[1] Vos T., Barber R. M., Bell B. (2015). Global, regional, and national incidence, prevalence, and years lived with disability for 301 acute and chronic diseases and injuries in 188 countries, 1990–2013: a systematic analysis for the Global Burden of Disease Study 2013. *The Lancet*.

[2] Saunders J. C., Dear S. P., Schneider M. E. (1985). The anatomical consequences of acoustic injury: a review and tutorial. *The Journal of the Acoustical Society of America*.

[3] Kiang N. Y., Moxon E. C., Levine R. A. (1970). Auditory-nerve activity in cats with normal and abnormal cochleas. *Ciba Foundation Symposium‐Sensorineural Hearing Loss*.

[4] Xu H., Fan W., Zhao X. (2016). Disrupted functional brain connectome in unilateral sudden sensorineural hearing loss. *Hearing Research*.

[5] Xia S., Song T., Che J. (2017). Altered brain functional activity in infants with congenital bilateral severe sensorineural hearing loss: a resting-state functional MRI study under sedation. *Neural Plasticity*.

[6] Peelle J. E., Troiani V., Grossman M., Wingfield A. (2011). Hearing loss in older adults affects neural systems supporting speech comprehension. *The Journal of Neuroscience*.

[7] Wang X., Fan Y., Zhao F. (2014). Altered regional and circuit resting-state activity associated with unilateral hearing loss. *PLoS One*.

[8] Puschmann S., Sandmann P., Bendixen A., Thiel C. M. (2014). Age-related hearing loss increases cross-modal distractibility. *Hearing Research*.

[9] Lin F. R., Metter E. J., O’Brien R. J., Resnick S. M., Zonderman A. B., Ferrucci L. (2011). Hearing loss and incident dementia. *Archives of Neurology*.

[10] Benetti S., van Ackeren M. J., Rabini G. (2017). Functional selectivity for face processing in the temporal voice area of early deaf individuals. *Proceedings of the National Academy of Sciences of the United States of America*.

[11] Westcott M. (2006). Acoustic shock injury (ASI). *Acta Oto-Laryngologica*.

[12] Baumann O., Borra R. J., Bower J. M. (2015). Consensus paper: the role of the cerebellum in perceptual processes. *The Cerebellum*.

[13] Holmes G. (1939). The cerebellum of man. *Brain*.

[14] Doyon J., Penhune V., Ungerleider L. G. (2003). Distinct contribution of the cortico-striatal and cortico-cerebellar systems to motor skill learning. *Neuropsychologia*.

[15] Huang C. M., Liu G., Huang R. (1982). Projections from the cochlear nucleus to the cerebellum. *Brain Research*.

[16] Sun X., Jen P. H. S., Kamada T. (1983). Mapping of the auditory area in the cerebellar vermis and hemispheres of the mustache bat, *Pteronotus parnellii parnellii*. *Brain Research*.

[17] Wang X. F., Woody C. D., Chizhevsky V., Gruen E., Landeira-Fernandez J. (1991). The dentate nucleus is a short-latency relay of a primary auditory transmission pathway. *Neuroreport*.

[18] Gacek R. R. (1973). A cerebellocochlear nucleus pathway in the cat. *Experimental Neurology*.

[19] Keifer O. P., Gutman D. A., Hecht E. E., Keilholz S. D., Ressler K. J. (2015). A comparative analysis of mouse and human medial geniculate nucleus connectivity: a DTI and anterograde tracing study. *NeuroImage*.

[20] Huffman R. F., Henson O. W. (1990). The descending auditory pathway and acousticomotor systems: connections with the inferior colliculus. *Brain research reviews*.

[21] Aitkin L. M., Boyd J. (1975). Responses of single units in cerebellar vermis of the cat to monaural and binaural stimuli. *Journal of Neurophysiology*.

[22] Snider R. S., Stowell A. (1944). Receiving areas of the tactile, auditory, and visual systems in the cerebellum. *Journal of Neurophysiology*.

[23] Wolfe J. W., Kos C. M. (1975). Cerebellar inhibition of auditory function. *Transactions. Section on Otolaryngology. American Academy of Ophthalmology and Otolaryngology*.

[24] Lockwood A. H., Salvi R. J., Coad M. L. (1999). The functional anatomy of the normal human auditory system: responses to 0.5 and 4.0 kHz tones at varied intensities. *Cerebral Cortex*.

[25] Chen Y. C., Li X., Liu L. (2015). Tinnitus and hyperacusis involve hyperactivity and enhanced connectivity in auditory-limbic-arousal-cerebellar network. *eLife*.

[26] Bauer C. A., Kurt W., Sybert L. T., Brozoski T. J. (2013). The cerebellum as a novel tinnitus generator. *Hearing Research*.

[27] Petacchi A., Kaernbach C., Ratnam R., Bower J. M. (2011). Increased activation of the human cerebellum during pitch discrimination: a positron emission tomography (PET) study. *Hearing Research*.

[28] Jiang C., Luo B., Manohar S., Chen G. D., Salvi R. (2017). Plastic changes along auditory pathway during salicylate-induced ototoxicity: hyperactivity and CF shifts. *Hearing Research*.

[29] Petacchi A., Laird A. R., Fox P. T., Bower J. M. (2005). Cerebellum and auditory function: an ALE meta-analysis of functional neuroimaging studies. *Human Brain Mapping*.

[30] Middleton F. A., Strick P. L. (2000). Basal ganglia and cerebellar loops: motor and cognitive circuits. *Brain Research Reviews*.

[31] Konarski J. Z., McIntyre R. S., Grupp L. A., Kennedy S. H. (2005). Is the cerebellum relevant in the circuitry of neuropsychiatric disorders?. *Journal of Psychiatry & Neuroscience*.

[32] Lane R. D., Reiman E. M., Ahern G. L., Schwartz G. E., Davidson R. J. (1997). Neuroanatomical correlates of happiness, sadness, and disgust. *The American Journal of Psychiatry*.

[33] Bonne O., Gilboa A., Louzoun Y. (2003). Resting regional cerebral perfusion in recent posttraumatic stress disorder. *Biological Psychiatry*.

[34] Wegiel J., Wisniewski H. M., Dziewiatkowski J. (1999). Cerebellar atrophy in Alzheimer’s disease—clinicopathological correlations. *Brain Research*.

[35] Miquel M., Toledo R., Garcia L. I., Coria-Avila G. A., Manzo J. (2009). Why should we keep the cerebellum in mind when thinking about addiction?. *Current Drug Abuse Reviews*.

[36] Gomaa M. A. M., Elmagd M. H. A., Elbadry M. M., Kader R. M. A. (2014). Depression, anxiety and stress scale in patients with tinnitus and hearing loss. *European Archives of Oto-Rhino-Laryngology*.

[37] Contrera K. J., Betz J., Deal J. (2017). Association of hearing impairment and anxiety in older adults. *Journal of Aging and Health*.

[38] Cosh S., von Hanno T., Helmer C. (2018). The association amongst visual, hearing, and dual sensory loss with depression and anxiety over 6 years: The Tromsø Study. *International Journal of Geriatric Psychiatry*.

[39] Jayakody D. M. P., Almeida O. P., Speelman C. P. (2018). Association between speech and high-frequency hearing loss and depression, anxiety and stress in older adults. *Maturitas*.

[40] Feng Y., Chen Y. C., Lv H. (2018). Increased resting-state cerebellar-cerebral functional connectivity underlying chronic tinnitus. *Frontiers in Aging Neuroscience*.

[41] Snider R. S. (1950). Recent contributions to the anatomy and physiology of the cerebellum. *Archives of Neurology and Psychiatry*.

[42] Huang C., Liu G. (1990). Organization of the auditory area in the posterior cerebellar vermis of the cat. *Experimental Brain Research*.

[43] Hribar M., Suput D., Carvalho A. A., Battelino S., Vovk A. (2014). Structural alterations of brain grey and white matter in early deaf adults. *Hearing Research*.

[44] Zhang Y., Mao Z., Feng S. (2018). Altered functional networks in long-term unilateral hearing loss: a connectome analysis. *Brain and Behavior: A Cognitive Neuroscience Perspective*.

[45] Lin F. R., Thorpe R., Gordon-Salant S., Ferrucci L. (2011). Hearing loss prevalence and risk factors among older adults in the United States. *The Journals of Gerontology: Series A*.

[46] World Medical Association (2013). World Medical Association Declaration of Helsinki: ethical principles for medical research involving human subjects. *JAMA*.

[47] Friston K. J., Williams S., Howard R., Frackowiak R. S. J., Turner R. (1996). Movement-related effects in fMRI time-series. *Magnetic Resonance in Medicine*.

[48] Yan C. G., Cheung B., Kelly C. (2013). A comprehensive assessment of regional variation in the impact of head micromovements on functional connectomics. *NeuroImage*.

[49] Ciric R., Wolf D. H., Power J. D. (2017). Benchmarking of participant-level confound regression strategies for the control of motion artifact in studies of functional connectivity. *NeuroImage*.

[50] Lin C. H., Chen C. M., Lu M. K. (2013). VBM reveals brain volume differences between Parkinson’s disease and essential tremor patients. *Frontiers in Human Neuroscience*.

[51] Seeley W. W., Menon V., Schatzberg A. F. (2007). Dissociable intrinsic connectivity networks for salience processing and executive control. *The Journal of Neuroscience*.

[52] Timmann D., Drepper J., Frings M. (2010). The human cerebellum contributes to motor, emotional and cognitive associative learning. A review. *Cortex*.

[53] Middleton F. A., Strick P. L. (2001). Cerebellar projections to the prefrontal cortex of the primate. *The Journal of Neuroscience*.

[54] Brozoski T., Brozoski D., Wisner K., Bauer C. (2017). Chronic tinnitus and unipolar brush cell alterations in the cerebellum and dorsal cochlear nucleus. *Hearing Research*.

[55] Daftary A., Shulman A., Strashun A. M., Gottschalk C., Zoghbi S. S., Seibyl J. P. (2004). Benzodiazepine receptor distribution in severe intractable tinnitus. *The International Tinnitus Journal*.

[56] Dobromyslin V. I., Salat D. H., Fortier C. B. (2012). Distinct functional networks within the cerebellum and their relation to cortical systems assessed with independent component analysis. *NeuroImage*.

[57] Buckner R. L., Krienen F. M., Castellanos A., Diaz J. C., Yeo B. T. T. (2011). The organization of the human cerebellum estimated by intrinsic functional connectivity. *Journal of Neurophysiology*.

[58] Zhang Y. T., Geng Z. J., Zhang Q., Li W., Zhang J. (2006). Auditory cortical responses evoked by pure tones in healthy and sensorineural hearing loss subjects: functional MRI and magnetoencephalography. *Chinese Medical Journal*.

[59] Deschamps I., Baum S. R., Gracco V. L. (2014). On the role of the supramarginal gyrus in phonological processing and verbal working memory: evidence from rTMS studies. *Neuropsychologia*.

[60] Oliveri M., Vallar G. (2009). Parietal versus temporal lobe components in spatial cognition: setting the mid-point of a horizontal line. *Journal of Neuropsychology*.

[61] Schraa-Tam C. K. L., Rietdijk W. J. R., Verbeke W. J. M. I. (2012). fMRI activities in the emotional cerebellum: a preference for negative stimuli and goal-directed behavior. *The Cerebellum*.

[62] Olson I. R., Plotzker A., Ezzyat Y. (2007). The enigmatic temporal pole: a review of findings on social and emotional processing. *Brain*.

[63] Terasawa Y., Fukushima H., Umeda S. (2013). How does interoceptive awareness interact with the subjective experience of emotion? An fMRI study. *Human Brain Mapping*.

[64] Pantazatos S. P., Talati A., Schneier F. R., Hirsch J. (2014). Reduced anterior temporal and hippocampal functional connectivity during face processing discriminates individuals with social anxiety disorder from healthy controls and panic disorder, and increases following treatment. *Neuropsychopharmacology*.

[65] Li W., Cui H., Zhu Z. (2016). Aberrant functional connectivity between the amygdala and the temporal pole in drug-free generalized anxiety disorder. *Frontiers in Human Neuroscience*.

[66] Stoodley C. J. (2012). The cerebellum and cognition: evidence from functional imaging studies. *The Cerebellum*.

[67] Wall N. R., De La Parra M., Callaway E. M., Kreitzer A. C. (2013). Differential innervation of direct- and indirect-pathway striatal projection neurons. *Neuron*.

[68] Belin P., McAdams S., Thivard L. (2002). The neuroanatomical substrate of sound duration discrimination. *Neuropsychologia*.

[69] Ristanovic D., Milosevic N. T., Stefanovic B. D., Maric D. L., Rajkovic K. (2010). Morphology and classification of large neurons in the adult human dentate nucleus: a qualitative and quantitative analysis of 2D images. *Neuroscience Research*.

[70] Meredith M. A., Clemo H. R., Corley S. B., Chabot N., Lomber S. G. (2016). Cortical and thalamic connectivity of the auditory anterior ectosylvian cortex of early-deaf cats: implications for neural mechanisms of crossmodal plasticity. *Hearing Research*.

[71] Lyness R. C., Alvarez I., Sereno M. I., MacSweeney M. (2014). Microstructural differences in the thalamus and thalamic radiations in the congenitally deaf. *NeuroImage*.

[72] Namkung H., Kim S. H., Sawa A. (2017). The insula: an underestimated brain area in clinical neuroscience, psychiatry, and neurology. *Trends in Neurosciences*.

[73] Craig A. D. (. B.). (2009). How do you feel — now? The anterior insula and human awareness. *Nature Reviews Neuroscience*.

[74] Golden H. L., Agustus J. L., Nicholas J. M. (2016). Functional neuroanatomy of spatial sound processing in Alzheimer’s disease. *Neurobiology of Aging*.

[75] Stein M. B., Simmons A. N., Feinstein J. S., Paulus M. P. (2007). Increased amygdala and insula activation during emotion processing in anxiety-prone subjects. *The American Journal of Psychiatry*.

[76] Cho Y. T., Fromm S., Guyer A. E. (2013). Nucleus accumbens, thalamus and insula connectivity during incentive anticipation in typical adults and adolescents. *NeuroImage*.

[77] Irvine D. R. F. (2018). Plasticity in the auditory system. *Hearing Research*.

[78] Eckstrand K. L., Hanford L. C., Bertocci M. A. (2018). Trauma-associated anterior cingulate connectivity during reward learning predicts affective and anxiety states in young adults. *Psychological Medicine*.

[79] Jin C., Li H., Li X. (2018). Temporary hearing threshold shift in healthy volunteers with hearing protection caused by acoustic noise exposure during 3-T multisequence MR neuroimaging. *Radiology*.

[80] Rubinov M., Sporns O. (2010). Complex network measures of brain connectivity: uses and interpretations. *NeuroImage*.

